# Role of Myofibrillar Protein Catabolism in Development of Glucocorticoid Myopathy: Aging and Functional Activity Aspects

**DOI:** 10.3390/metabo6020015

**Published:** 2016-05-13

**Authors:** Teet Seene, Priit Kaasik

**Affiliations:** Institute of Sport Sciences and Physiotherapy, University of Tartu, Ravila 14a, Tartu 50411, Estonia; priit.kaasik@ut.ee

**Keywords:** glucocorticoid myopathy, activity of daily living, fiber types, myofibrils, protein catabolism, aging, exercise

## Abstract

Muscle weakness in corticosteroid myopathy is mainly the result of the destruction and atrophy of the myofibrillar compartment of fast-twitch muscle fibers. Decrease of titin and myosin, and the ratio of nebulin and MyHC in myopathic muscle, shows that these changes of contractile and elastic proteins are the result of increased catabolism of the abovementioned proteins in skeletal muscle. Slow regeneration of skeletal muscle is in good correlation with a decreased number of satellite cells under the basal lamina of muscle fibers. Aging causes a reduction of AMP-activated protein kinase (AMPK) activity as the result of the reduced function of the mitochondrial compartment. AMPK activity increases as a result of increased functional activity. Resistance exercise causes anabolic and anticatabolic effects in skeletal muscle: muscle fibers experience hypertrophy while higher myofibrillar proteins turn over. These changes are leading to the qualitative remodeling of muscle fibers. As a result of these changes, possible maximal muscle strength is increasing. Endurance exercise improves capillary blood supply, increases mitochondrial biogenesis and muscle oxidative capacity, and causes a faster turnover rate of sarcoplasmic proteins as well as qualitative remodeling of type I and IIA muscle fibers. The combination of resistance and endurance exercise may be the fastest way to prevent or decelerate muscle atrophy due to the anabolic and anticatabolic effects of exercise combined with an increase in oxidative capacity. The aim of the present short review is to assess the role of myofibrillar protein catabolism in the development of glucocorticoid-caused myopathy from aging and physical activity aspects.

## 1. Introduction

**Figure 1:**
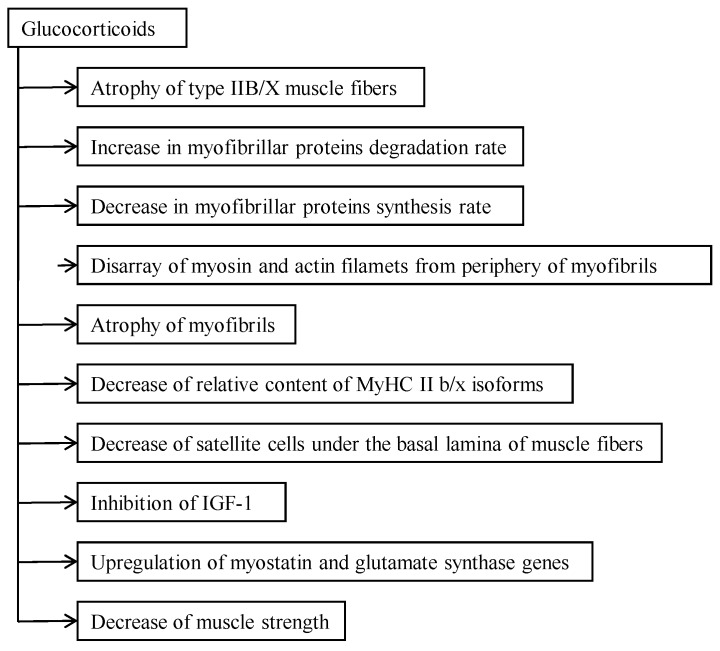
Effect of glucocorticoids on skeletal muscle.

Glucocorticoids, well-known immunosuppressive and anti-inflammatory drugs, have unfortunate side effects upon long-term use and high doses: fast twitch (FT) muscle fiber atrophy and development of myopathy [1,2]. Glucocorticoids cause reduction in muscle mass, selective atrophy of FT muscle fibers, wasting of muscle, and loss of strength (Figure 1). Glucocorticoids increase the expression of myostatin, a negative regulator of skeletal muscle growth. The changes described above appeared particularly in FT muscle fibers [3], including changes in the turnover rate of muscle proteins [4,5]. Major changes take place in the myofibrillar compartment [6] and in the turnover rate of two main contractile proteins, myosin and actin [7,8,9]. So, myofibrils of glucocorticoids caused myopathic IIB muscle fibers (fibers with low oxidative capacity) to show disarray of myosin myofilaments, increased proteolytic activity [10], a decreased synthesis rate of myosin and actin [9], and a decrease in myosin-heavy chain (MyHC) IIb isoforms’ relative content [11]. Reduced muscle elasticity, increased tone, and stiffness with a concomitant decrease in cytoskeletal proteins titin and nebulin and contractile protein myosin content are accompanied by muscle atrophy [12]. Both glucocorticoids caused myopathic and aging-caused sarcopenic muscle wasting is mainly a result of decrease of FT muscle fibers and atrophy of these fibers [13]. In both cases it is mainly the myofibrillar proteins synthesis rate that decreases, but not the sarcoplasmic proteins [10,14]. Both sarcopenic and glucocorticoids caused myopathic muscles to have impaired locomotion, general weakness, and diminished capacity for regeneration [15,16]. Slow regeneration of skeletal muscle is in good correlation with a decreased number of satellite cells under the basal lamina of muscle fibers [15]. The catabolic action of glucocorticoids and aging on skeletal muscle also depend on the functional activity of muscle [7,17,18]. Therefore the aim of the present short review was to assess the role of myofibrillar protein catabolism in the development of glucocorticoid-caused myopathy from aging and functional activity aspects.

## 2. The Catabolic Action of Glucocorticoids

Glucocorticoids, chemically classed as steroids, are hormones that affect the metabolism of carbohydrates. These steroids have an anti-inflammatory effect, but there are also many side effects of catabolic action. The catabolic effect of glucocorticoids depends on individual tissues. In contrast to the loss of protein from skeletal muscle, bone, lymphoid tissue, and smooth muscle, there is an increase in the rate of cardiac, hepatic, and urogenital activities [1]. The catabolic action of glucocorticoids depends on the type of muscle fibers [7]. FT muscles, particularly type IIB fibers, which have the lowest oxidative capacity among the striated muscle fibers, are most sensitive to the catabolic action of glucocorticoids (Figure 1). In these fibers the activity of non-lysosomal proteases increases significantly after glucocorticoid administration [7]. Large doses of glucocorticoids depress testosterone and insulin levels [8,19], although the inhibition of protein synthesis after glucocorticoid administration plays a lesser role in the atrophy of muscle fibers than accelerated protein catabolism [1]. As the content of lysosomes in type IIB/X muscle fibers is low, the role of lysosomal proteases in the development of corticosteroid myopathy is not significant. It has been shown that the process of atrophy starts from these myosin filaments located in the periphery of myofibrils. Myosin filaments lysed after separation [10], while actin filaments are more resistant to the catabolic action of glucocorticoids. Muscle weakness in corticosteroid myopathy is mainly the result of destruction and atrophy of the myofibrillar compartment of skeletal muscle [6]. We have to agree with Kelly *et al.* [20], who about three decades ago stated that the terms catabolic and myopathic should be used carefully as they can be misleading descriptions of the action of glucocorticoids on the level of the organism as well as on the level of skeletal muscle. Disappearance of about 20 percent of myosin filaments from myofibrils of muscle fibers with low oxidative capacity and decrease of MyHC IIb isoform relative content [6] are the explanation for decreased muscle strength and motor activity in the case of corticosteroid myopathy. This is qualitative remodeling of the myofibrillar compartment of myopathic type II B/X fibers but not whole skeletal muscle. The higher the degree of atrophy, the lower the muscle elasticity and the higher the tone. Muscle tone is dependent on changes in innervation. It has been shown that glucocorticoid myopathic FT muscles’ neuromuscular synapses are destroyed [2]. A decrease of titin and myosin [21] and of the ratio of nebulin and MyHC in myopathic muscle [12], shows that these changes in contractile and elastic proteins are the result of elevated catabolism of the abovementioned proteins in skeletal muscle. This is the reason for reduced elasticity and generation of tension in glucocorticoid-caused myopathic muscle.

### 2.1. Relationship between Catabolic Action and Functional Activity of Muscle

More than four decades ago it was shown that the catabolic action of corticosteroids depends on the state of functional activity of skeletal muscle [4]. Moderate endurance type exercise has been shown to be effective in retarding muscle atrophy [17] and protecting against wasting [22]. The effect of moderate exercise in inducing a less pronounced catabolic effect of glucocorticoid is caused by the elevation of anticatabolic activity of this type of exercise and related with the endogenous action of androgens in the stimulation of anticatabolic activity in type IIB/X muscle fibers [7]. Skeletal muscles with higher oxidative capacity, particularly slow twitch (ST) fibers, are less sensitive to the catabolic action of glucocorticoids; this phenomena was explained by the lesser elevation of proteolytic activity in these muscle fibers, but in muscle fibers where oxidative capacity is low, the catabolic activity was more pronounced [10]. As ST muscle fibers are involved in the maintenance of static body posture, in slow repetitive movements, and being functionally active even when FT fibers are passive, it may be also explain why there is no significant catabolic action of glucocorticoids and atrophy of ST muscle fibers [10]. Atrophy of muscle fibers with low oxidative capacity is the result of inhibition of insulin-like growth factor-1 (IGF-1) [23] and upregulation of two genes, myostatin and glutamate synthase [24]. Increased functional activity in the elderly improves glucose intolerance and insulin signaling, reduces tumor necrosis factor-*α*, increases adiponectin and IGF-1 concentrations, and reduces total and abdominal visceral fat [25]. Increase of muscle activity increases the synthesis rate of myofibrillar proteins [26] via a mammalian target of rapamycin-activating proteins within the nitrogen-activated protein kinase signaling [27]. The recovery from the last exercise session, particularly from intensive exercise, is faster in the young than in the elderly [28].

### 2.2. Aging Aspect of Catabolic Action of Glucocorticoids

**Figure 2:**
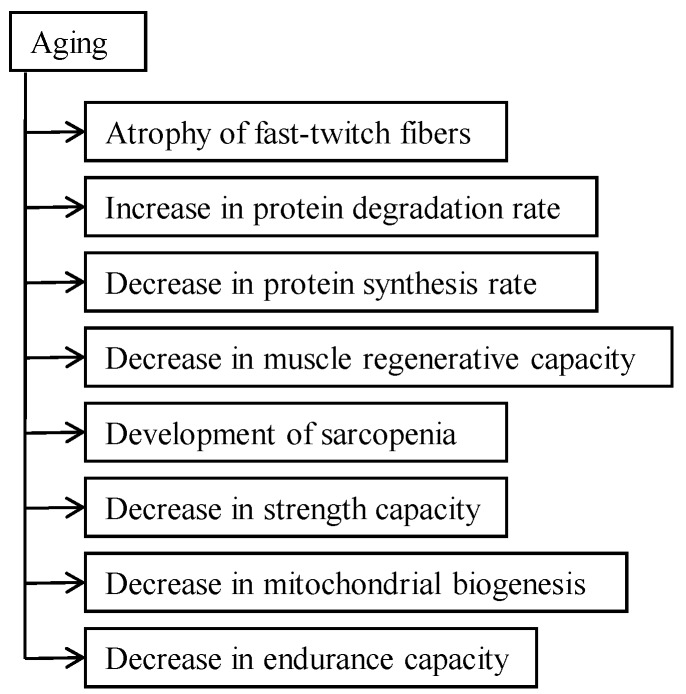
Effect of aging on skeletal muscle.

Over a lifespan there is a decrease of skeletal muscle mass, primarily as a result of the reduction of FT fibers (Figure 2), accompanied by deterioration of muscle quality [29,30]. The possible regeneration of skeletal muscle in the elderly [31] depends on the decrease of satellite cells [32]. Old glucocorticoid-caused myopathic rats have only one half the number of satellite cells that young ones do. Despite that skeletal muscle regeneration, although the adipose tissue content was significantly higher in old myopathic rats and muscle the strength decrease was about 50% [15]. In glucocorticoid-treated aged rats muscle wasting was more rapid than in young ones and recovery of muscle mass took twice as long as in the young [33]. The reason for that is the decrease of the stimulatory effect of insulin and IGF-1 in the skeletal muscle of old rats, which is twice as severe in the young [34]. Aging-caused sarcopenia and glucocorticoid-caused myopathy both develop as a result of the decrease and damage of satellite cells [35,36]. In old myopathic muscle, degradation of contractile proteins doubles [15]. Many intrinsic changes in cells accompany aging: accumulation of oxidative damage, decline in genome maintenance, and diminished mitochondrial function [37,38]. These changes may induce muscle destruction and therefore delay the regeneration of the myofibrillar compartment. Increased functional activity of muscle tissue causes fast recovery of muscle contractile structures and strength and depends on the oxidative capacity of muscle [39]. Contractile proteins’ synthesis rate is more intensive in muscle fibers with a high oxidative capacity [40,41]. A decrease in AMP-activated protein kinase (AMPK) activity in the elderly is the reason for reduced mitochondrial function. AMPK is activated in response to moderate functional activity and may be an effective measure in the prevention of disability and diseases in the elderly [18].

## 3. Catabolic Action of Glucocorticoids in the Myofibrillar Compartment

Muscle fibers and myofibrils of glucocorticoid-caused myopathic glycolytic muscle are thinner in comparison with the control group and disappeared completely from one fifth of the area of myofibrils of myopathic muscle myosin filaments [6]. The intensive destruction of myofibrils and degradation of contractile proteins, including MyHC IIb isoform [42], are the main reasons for reduced muscle strength, motor activity, and weakness in glucocorticoid-caused myopathic rats [11,15]. Destruction of myofibrils starts from the periphery of glycolytic muscle fibers, from myosin filaments (Figure 1), and spreads all over the myofibrillar compartment [10]. The second reason why myofibrils of myopathic muscle fibers are thinner is the slower myofibrillar protein synthesis rate and assembly of filaments. The decrease of the MyHC IIb isoform relative content and the increase of the MyHC IId isoform show that the quantitative changes in myofibrils are significantly related to the qualitative remodeling of thick myofilaments in myopathic glycolytic muscle fibers [11]. Changes in the myofibril ultrastructure of myopathic muscle fibers are also related to the functional modification of glycolytic muscle fibers. These modifications were not observed in muscle fibers with higher oxidative capacity in myopathic rodents [11]. Glucocorticoids caused wasting in senescent and young rodents as a result of the loss of FT fibers, their myofibrils, contractile proteins, and conversion of muscle fibers with low oxidative capacity into higher oxidative capacity [11,13]. The myosin HC and actin synthesis rate in aging rats decreased by about 20–30% and contractile proteins turned over in old subjects very slowly; the same tendency continued after glucocorticoid treatment [1].

## 4. Effect of Glucocorticoids on Muscle Strength and Motor Activity

During ageing muscle strength decreases significantly; for example, the hindlimb grip strength in old rats decreases by about 50% due to sarcopenia [15]. Glucocorticoid treatment decreases muscle strength in both young and old groups. In the old group the decrease was more significant than in the young. Ageing is accompanied by general weakness and impaired locomotion [14]. Motor activity in old rats decreased in comparison with the young group and glucocorticoid treatment reduced it in both age groups [15]. An excess of glucocorticoids decreases the skeletal muscle regeneration capacity in the young and old groups and is in correlation with a decrease of satellite cells under the basal lamina of skeletal muscle fibers [1,43]. The intensity of the regeneration of skeletal muscle depends on the mass of muscle and contractile properties of muscle [44]. Glucocorticoid-caused myopathy induces structural changes in satellite cells’ ultrastructure [10]. These structural changes in satellite cells are similar to the changes in skeletal muscle fibers’ structure. A decrease in satellite cells and changes in their structure are the reasons for decreased regeneration capacity of age-related and glucocorticoid-caused myopathic muscle. Viscoelastic properties illustrate skeletal muscle function in physiopathological conditions. There is a positive correlation between muscle atrophy and elasticity and a negative correlation between state of atrophy and muscle tone [12]. A decrease in contractile protein myosin and in elastic proteins titin and nebulin leads to the reduction of muscle elasticity and generation of tension in myopathic muscle [12]. A decrease in the oxidative capacity and contractile properties in elderly and myopathic muscle are the reasons for changes in muscle quantity and quality, which lead to disability.

### 4.1. Glucocorticoid-Caused Myopathy, Sarcopenia, and Cachexia

Protein degradation, particularly myofibrillar protein degradation, is typical for glucocorticoid myopathy. Protein degradation in skeletal muscle fibers, particularly in FT fibers with low oxidative capacity, is mediated by the activity of the ubiquitin–proteosomal and the lysosomal pathways [45]. The activity of the ubiquitin–proteosomal pathway is significantly increased in atrophying muscle due to transcriptional activation of E3 ligase-encoding genes atrogin-1 and MuRF 1 [46]. A glucocorticoid receptor in skeletal muscle (REDD1, KLF15) inhibits mTOR activity via BCAT 2 gene activation. KLF15 upregulates the expression of E3 ubiquitin ligases atrogin-1 and MuRF 1, which cause the muscle fiber to atrophy [46]. Cachexia is usually accompanied by the loss of body mass but not sarcopenia and muscle wasting, and is associated with reduction of exercise capacity and reduced quality of life [47]. The loss of muscle in cachexia is the imbalance between protein synthesis and degradation. The ubiquitin–proteasome pathway, satellite cells in muscle, and the related receptors function and signaling pathways influence this process by tumor-induced systematic inflammation [48]. Glucocorticoid myopathy, muscle wasting, and involuntary loss of muscle quantity, known as cachexia, are great challenges in clinical practice. Research into this area of anti-inflammatory pathways and anabolics continues with trials to improve skeletal muscle mass and function [49]. Chrelin influences food intake and the release of growth hormone; it inhibits the production of tumor necrosis factor and the proinflammatory cytokines IL-1α, IL-6, and induces the anti-inflammatory cytokine IL-10 [47]. In the case of patients suffering from chronic illness the terms myopenia and muscle-wasting disease have been proposed for use [48,50]. During the last year the use of the term sarcopenia has been suggested for the case of the healthy elderly who lose muscle mass during ageing [49].

In the case of corticosteroid myopathy, sarcopenia, and cachexia, the molecular mechanisms of regulation of muscle, metabolism, and induced wasting disorder-related skeletal muscle atrophy is almost similar. Cachexia is characterized by the loss of adipose tissue as a result of an imbalance in lipogenesis and lipolysis, with enhanced lipolysis supported by neuroendocrine activation and tumor-related lipolytic factors [51].

### 4.2. How to Prevent or Decelerate Skeletal Muscle Atrophy and Disability

**Figure 3:**
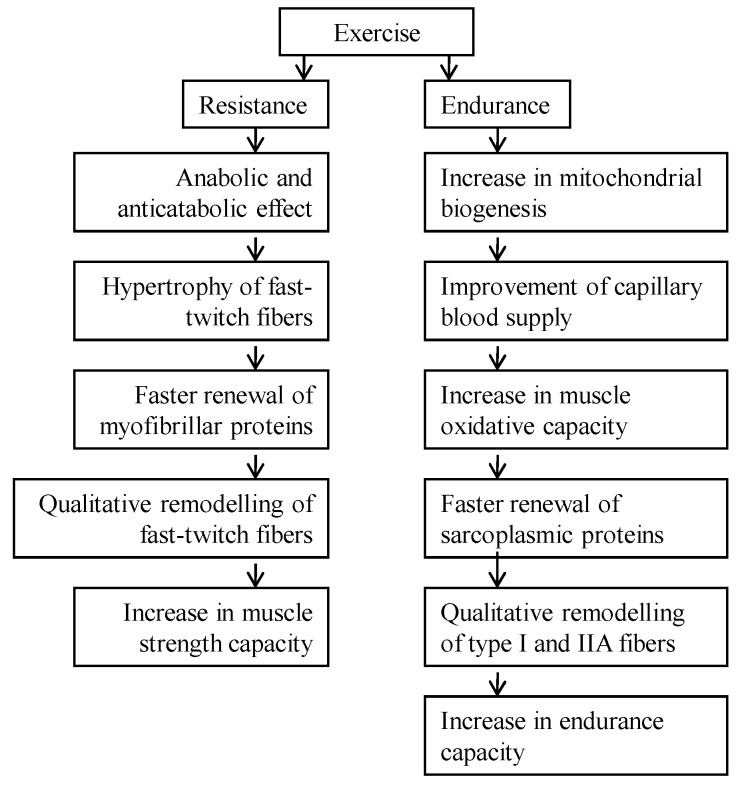
Effect of increased functional activity on skeletal muscle.

The central question is how to prevent or decelerate skeletal muscle atrophy in the elderly and glucocorticoid myopathic patients. Aging causes a reduction of AMPK activity as the result of reduced function of the mitochondrial compartment [52]. AMPK activity increases as a result of increased functional activity in fast twitch muscle fibers [53]. At the same time it has been shown that in physiological conditions AMPK may block the activation of mammalian target of rapamycin complex-1 (TORC 1) via phosphorylation and activation of the tuberous sclerosis complex-2 (TSC 2) [54]. It is not clear yet in what conditions (character of muscle activity, duration of activity, and recovery period between activities) AMPK blocks the activation of TORC 1. The optimization of increased functional activity programs that lead to interaction between signaling pathways in myofibrillar and mitochondrial compartments (protein synthesis/degradation rate) has proven to be effective in the elderly and myopathic population at causing muscle hypertrophy and maintaining endurance capacity at the same time [18]. Resistance type muscle activity causes anabolic and anticatabolic effects in skeletal muscle: FT muscle fibers hypertrophy and myofibrillar proteins turn over faster. These changes are leading to the qualitative remodeling of FT muscle fibers (Figure 3). As a result of these changes, muscle strength is increasing [18]. Endurance-type muscle activity improves capillary blood supply, increases mitochondrial biogenesis and muscle oxidative capacity, and causes a faster turnover rate of sarcoplasmic proteins as well as qualitative remodeling of type I and IIA muscle fibers (Figure 3) [18]. As both muscle strength and endurance decrease in the elderly, particularly during glucocorticoid administration, a combination of endurance and resistance training may give the best rehabilitative effect. This combination may prevent or decelerate muscle atrophy due to the anabolic and anticatabolic effects of exercise. It is not yet known whether and in what conditions AMPK blocks the activation of TORC 1 by activating TSC 2 during concurrent strength and endurance exercise in these physiopathological conditions. The problem may be the ability of muscle fibers in conditions of glucocorticoid-caused myopathy and sarcopenia to hypertrophy and increase or maintain the endurance capacity at the same time. Theoretically, concurrent endurance and resistance exercise is the fastest way to decelerate sarcopenia and the decrease of muscle strength in the elderly as well as in glucocorticoid-caused myopathic muscle. In practice, concurrent endurance and resistance training in the elderly population has been shown to increase muscle strength, endurance, and cardiorespiratory fitness [18].

## 5. Conclusions

Muscle weakness in corticosteroid myopathy is the result of destruction and atrophy of fast-twitch muscle fibers and their myofibrillar machinery. The disappearance of one fifth of myosin filaments from myofibrils and a decrease in MyHC IIb isoform relative content are the reasons for decreased muscle strength and motor activity in the case of corticosteroid myopathy. The higher the degree of muscle atrophy, the lower the muscle elasticity and the higher the tone. A decrease in titin and myosin, and in the ratio of nebulin and MyHC in myopathic muscle, shows that these changes of contractile and elastic proteins are the result of increased catabolism of these proteins in skeletal muscle. Both glucocorticoid-caused myopathic and aging-caused sarcopenic muscle wasting is mainly a result of the decrease and atrophy of fast-twitch muscle fibers. In both cases, the myofibrillar protein synthesis rate decreases. Slow regeneration of skeletal muscle is in good correlation with a decreased number of satellite cells under the basal lamina of muscle fibers. Aging causes a reduction of AMPK activity as the result of reduced function of the mitochondrial compartment. AMPK activity increases as a result of increased functional activity. Resistance-type muscle activity causes anabolic and anticatabolic effects in skeletal muscle: muscle fibers hypertrophy and myofibrillar proteins turn over faster. These changes are leading to the qualitative remodeling of fast-twitch muscle fibers. As a result of these changes, muscle strength is increasing. Endurance-type muscle activity improves capillary blood supply, increases mitochondrial biogenesis and muscle oxidative capacity, and causes a faster turnover rate of sarcoplasmic proteins as well as qualitative remodeling of type I and IIA muscle fibers. As both muscle strength and endurance decrease in the elderly, particularly during glucocorticoid administration, the combination of endurance and resistance training may have the best rehabilitative effect.
